# Metals in *Calluna vulgaris*, *Empetrum nigrum*, *Festuca vivipara* and *Thymus praecox* ssp. *arcticus* in the geothermal areas of Iceland

**DOI:** 10.1007/s11356-021-15046-3

**Published:** 2021-07-10

**Authors:** Adam Rajsz, Bronisław Wojtuń, Aleksandra Samecka-Cymerman, Paweł Wąsowicz, Lucyna Mróz, Andrzej Rudecki, Alexander J. Kempers

**Affiliations:** 1 Department of Ecology, Biogeochemistry and Environmental Protection, Wrocław University, ul. Kanonia 6/8, 50-328 Wrocław, Poland; 2 Icelandic Institute of Natural History, Akureyri, Iceland; 3 Department of Environmental Science, Institute for Water and Wetland Research, Radboud University, Huygens Building, Heijendaalseweg 135, 6525 Nijmegen, AJ Netherlands

**Keywords:** Trace element, Bioindication, Low Arctic

## Abstract

This investigation was conducted to identify the content of metals in *Calluna vulgaris* (family *Ericaceae*), *Empetrum nigrum* (family *Ericaceae*), *Festuca vivipara* (family *Poaceae*) and *Thymus praecox* subsp. *arcticus* (family *Lamiaceae*), as well as in the soils where they were growing in eight geothermal heathlands in Iceland. Investigation into the vegetation of geothermal areas is crucial and may contribute to their proper protection in the future and bring more understanding under what conditions the plants respond to an ecologically more extreme situation. Plants from geothermally active sites were enriched with metals as compared to the same species from non-geothermal control sites (at an average from about 150 m from geothermal activity). The enriched metals consisted of Cd, Co, Cu, Fe and Ni in *C. vulgaris*; Cd, Mn and Ti in *E. nigrum*; Hg and Pb in *F. vivipara*; and Cd, Fe and Hg in *T. praecox*. Notably, *C. vulgaris*, *E. nigrum*, *F. vivipara* and *T. praecox* had remarkably high concentrations of Ti at levels typical of toxicity thresholds. Cd and Pb (except for *C. vulgaris* and *F. vivipara*) were not accumulated in the shoots of geothermal plants. *C. vulgaris* from geothermal and control sites was characterised by the highest bioaccumulation factor (BF) of Ti and Mn; *E. nigrum* and *F. vivipara* by the highest BF of Ti and Cr; and *T. praecox* by the highest BF of Ti and Zn compared to the other elements. In comparison with the other examined species, *F. vivipara* from geothermal sites had the highest concentration of Ti in above-ground parts at any concentration of plant-available Ti in soil.

**Supplementary Information:**

The online version contains supplementary material available at 10.1007/s11356-021-15046-3.

## Introduction

Iceland, an island situated in the Atlantic Ocean, has areas of volcanic activity from southwest to northeast, and geothermal areas occur (Elmarsdóttir et al. 2015). Such zones are of various sizes, elevation, climatic conditions and geothermal activity. Thus, geothermal activity is common in Iceland, but the associated unique environment (frequently remaining pristine) at the surface is usually small and occurs in patches. Such zones produce a very special stress-inducing environment and microclimatic conditions for plant development being less dependent on the climate (Burns and Leathwick 1995). They are characterised by high soil temperatures, higher humidity from steam evaporating vents and a specific pH range from acidic to alkaline accompanied by elevated levels of metals and metalloids, especially As and Ti (Glime 2007; Chiarucci et al. 2008; Wilberscheid 2008; Boothroyda 2009). According to Stout and Al-Niemi (2002) and Nishar et al. (2017), high soil temperatures seem to be the most important factor that determines the types of plant species in such areas. The stably elevated temperatures are additionally advantageous for plants of the geothermal zones in a cold climate (Wilberscheid 2008). Regular availability of moisture in such habitats with low natural water retention and low amounts of organic matter may produce special conditions for plants to survive. Additionally light intensity in the Nordic latitudes is probably an important factor owing to which vegetation can withstand the heat. The symbiotic fungi which provide nutrient supply produce melanin for complexing oxygen radicals. This activate systems responding to stress more quickly than in plants with no mycorrhiza and also provide protection for plants to cope with the high temperatures of geothermal zones (Redman et al. 2002). The vegetation systems of the Icelandic geothermal zones are yet to be fully investigated. The parameters of interest are usually soil temperature, pH and organic carbon concentration (Wilberscheid 2008; Elmarsdóttir et al. 2015). These reports do not discuss the influence of biogeochemical features on species distribution. Data about the level of rare minerals in plants and soils in Iceland could not be found in the literature. Therefore, this investigation was conducted to identify the content of potentially contaminating elements As, Cd, Co, Cr, Cu, Fe, Hg, Mn, Ni, Pb, Ti and Zn in *Calluna vulgaris* (L.) Hull, *Empetrum nigrum* L., *Festuca vivipara* (L.) Sm. and *Thymus praecox* subsp. *arcticus* (Durland) Jalas, abridged in this article as *T. praecox* (Wąsowicz 2020). The same minerals were determined in the soils where the plants were growing in eight geothermal heathlands in Iceland (Ottósson et al. 2016) as well as in the control sites surrounding the heathlands and not influenced by geothermal activity. *C. vulgaris* is a pioneering and one of the most widespread species able to survive in the harsh environmental conditions of waste heaps or geothermal fields with a range of pH of 2.0–7.0 and in nutrient-poor soils with temperatures as high as 55 °C (Engelskjøn et al. 2003; Chiarucci et al. 2008; Marrs and Bannister 2006; Lottermoser et al. 2011; Bartoli et al. 2013; Pippucci et al. 2015). *E. nigrum* is a species with a broad ecological amplitude that tolerates various types of substrates with pH of 2.5–7.7 (Monni et al. 2000; Monschein et al. 2010). It has been found in acidic habitats of Norway and has been reported to be able to detoxify or avoid uptake of, for example, excessive Al (Gjengedal et al. 2015). The species, tolerant to metals and surviving in very polluted sites, e.g. in the vicinity of smelters, is able to concentrate high levels of Cu and Ni (Monni et al. 2000; Monni et al. 2001; Zverev et al. 2008). *F. vivipara* belongs to a genus with a potential for evolving genetic adaptations in response to environmental conditions and especially elevated levels of metals and metalloids (Dradrach et al. 2020). This species has been found in Icelandic geothermal areas with soil temperatures of 15–98 °C (Elmarsdóttir et al. 2015). *T. praecox* grows in various sites such as gravelly soils, lava fields and meadows also including geothermal zones from North to Central Europe, Eastern North America, Greenland and Iceland (Pigott 1955; Kristinsson 2010). The tested hypothesis was that plants from geothermal zones contain significantly higher concentrations of metals than identical species from surrounding control sites away from high soil temperatures. Future protection of vegetation in geothermal areas is important, and this investigation enables better understanding on how plants in these areas respond to more extreme environmental factors.

## Materials and methods

### Sampling design

**Fig. 1:**
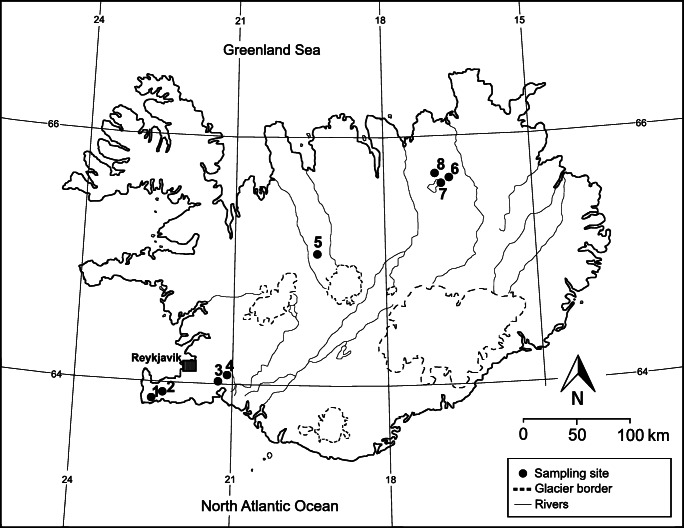
Location of the sampling sites

Samples were collected in heathland sites 1–8 (Fig. 1) influenced by geothermal activity and in nearby control sites without geothermal activity (at an average distance of about 150 m from geothermal activity) (Bartoli et al. 2013). It was not possible to separately mark the locations of geothermal and corresponding control sites with the scale of Fig. 1. Thus, sampling site numbers refer to both geothermal and their corresponding control sites. In each geothermal and control site, 3 squares of 25 m × 25 m were selected randomly, and in each square, above-ground biomass of *C. vulgaris* (except for geothermal sites 4 and 5 and control sites 2–5), *E. nigrum* (except for geothermal site 3), *F. vivipara* (except for control sites 6–8) and *T. praecox* (except for control sites 6 and 7) together with the topsoil (0–15-cm depth) was collected in 3 replicates. Plant samples were not washed (Aboal et al. 2011; Oliva and Valdés 2004). Samples were collected within the continental subarctic climate (*Dfc*) (Peel et al. 2007). Soil types were Brown Andosol (sites 1, 3–5), Leptosol (site 2) and Cambic Vitrisol-Sandy Vitrisol (sites 6–8) (Arnalds and Óskarsson 2009).

### Soil and plant analysis

Soil temperature was measured at a depth of 10 cm with a Pt-101 electronic thermometer (Elmetron). Soil samples previously cleaned from organic matter were passed through a 2-mm sieve (Morek Multiserw LPzE-2e). The total content of trace elements was analysed in soils homogenised in a Fritsch Pulverisette 2 mortar grinder, and the content of plant-available trace elements was determined in soils homogenised in a mortar with pestle. Plant-available concentrations (mg kg^-1^) of As, Cd, Co, Cr, Cu, Fe, Mn, Ni, Pb, Ti and Zn in soil were analysed by extraction with 1 M ammonium acetate-EDTA (pH 4.65) for 30 min (5 g dry soil in 50 mL) (Cottenie et al. 1982). Total concentrations of metals were analysed in soil and plant samples digested with HNO_3_ (65% ultra-pure) and H_2_O_2_ (70% ultra-pure) in a Speedwave Xpert Berghof microwave oven. Deionised water was used for diluting the digests. Fe, Mn, and Zn levels were detected by FAAS (Flame Atomic Absorption Spectrometry, Avanta PM GBC) and those of As, Cd, Co, Cr, Cu, Ni, Pb and Ti by GFAAS (Perkin-Elmer PinAAcle 900Z Graphite Furnace Atomic Absorption Spectrometry). The elements were detected against atomic absorption standard solution (Sigma Chemical Co.) and blanks of the same matrix as the samples. Element concentrations were calculated on a dry weight basis. Mercury was determined in powdered soil and plant samples by an AMA 254 Advanced Mercury Analyser. Accuracy of all elements was controlled with chestnut soil, Bainaimao and Bayan Obo, Neil Mongol in China GBW07402 (GSS-2) and Poaceae (mixture) IPE 952WEPAL Certified Reference Materials and presented in ESM 1 and ESM 2. All analyses were performed in triplicate.

### Statistical analysis

Normality and homogeneity of variance of the data were controlled with Shapiro-Wilk’s W and Brown-Forsythe tests (Argaç 2004). Because normal distribution was not detected for most of the examined elements, non-parametric tests were applied. Differences in altitude, temperature, pH and metal contents in soils and in *C. vulgaris*, *E. nigrum*, *T. praecox* and *F. vivipara* between geothermal and control sites were compared with a Mann-Whitney U test (P < 0.05). Multiple comparisons of mean ranks for soils and plants were applied to evaluate differences between the species within geothermal and control sites (P < 0.05).

Pearson correlation was applied for data with normal distribution.

To compare Ti accumulation from soil by *C. vulgaris*, *E. nigrum*, *F. vivipara* and *T. praecox*, an ANCOVA model was applied. In this model, the independent variable (covariate) was the Ti content available in soil, the dependent variable was Ti content in plants and the categorical predictor variable was each species. The interaction between the species and Ti concentration in soil tested the hypothesis whether the slopes of regression lines were equal between the species. If the slopes were not significantly different, we further tested whether the intercepts were equal. When significant differences were found, multiple comparison among intercepts was determined by the Tukey test (Zar 1999). A significance level of P < 0.05 was used throughout the study.

The bioaccumulation factor (BF) was calculated by dividing the element concentration in above-ground parts of *C. vulgaris*, *E. nigrum*, *T. praecox* and *F. vivipara* by the available element concentration in soil (Kandziora-Ciupa et al. 2017).

Statistica from Dell Inc (2016) was used for all calculations.

## Results and discussion

### Soil

Geothermal soils did not differ from control soils in altitude, but their temperature was significantly higher with more alkaline pH (U Mann Whitney test) than control sites (ESM 3). The maximum total metal contents for Icelandic soil presented by Panek and Kepinska (2002) were (mg kg^-1^) Cd 0.2–1.6, Cu 52–144, Pb 2.8–11 and Zn 65–124. In comparison with these data, the Cd content in the examined soil was lower and those of Cu, Pb and Zn were higher. The maximum concentrations of Co, Cr, Cu, Fe, Hg, Mn and Zn in geothermal sites were higher than those reported by Kolon et al. (2020) for Icelandic soil (ESM 3). The maximum total As concentration found was higher than the allowable level of 20 mg kg^-1^ in agricultural soil (Kabata-Pendias 2001). The Hg content of the control sites was within the average range of 0.05–0.3 mg kg^-1^ given by Kabata-Pendias (2001) and within the range of 0.01 to 0.3 mg kg^-1^ reported by Pastrana-Corral et al. (2016) for Mexican geothermal sites. The Hg content in the investigated geothermal sites was higher (ESM 3) than the above-mentioned values**.** The maximum total concentration of Ti in the examined soil of geothermal sites was lower than the level of 26,000 mg kg^-1^ reported in a similar area of New Zealand (Given 1980) but higher than 3300 mg kg^-1^ given by Kabata-Pendias (2001) as a global average for soils. Soils from geothermal sites contained significantly more (U Mann-Whitney test) total As, Cr, Fe, Hg, Ti and Zn than control soils (ESM 3). This is in agreement with Wilberscheid (2008) and Boothroyda (2009) that areas with geothermal activity are usually enriched with metals, especially As and Ti. Plant-available (DTPA) soil concentrations in Icelandic geothermal zones (ESM 4) were higher than reported by Burns and Leathwick (1995) for a similar area in New Zealand (in mg kg^-1^): Cu 0.05–0.7, Fe 31–153, Mn 2.5–7.5 and Zn 0.2–1.5. These authors report that levels of Cu and Zn in such areas are rather low, those of Mn are moderate and those of Fe are high with a tendency to increase with declining soil temperature. In this investigation, there was no significant difference in the concentration of available Fe in soil between geothermal and control zones (Burns and Leathwick 1995). Plant-available soil metal concentrations were lower (except for Mn) than those presented in ESM 4 for soil samples from other non-geothermal areas in Iceland (Kolon et al. 2020). Soils from the control sites in Iceland contained significantly more (U Mann-Whitney test) plant-available As, Cu, Ni and Ti and significantly less Mn than those from geothermal sites (ESM 4). This phenomenon was probably caused by low quantities of organic material in the geothermal soils (Wilberscheid 2008). Increased organic matter levels in the soil are related to lower availability of, for example, Mn (Graham et al. 1988).

### Plants

The maximum mass fractions of Cr, Cu, Fe and Ni in *C. vulgaris* (Table 1) from Iceland were higher and those of Cd, Mn, Pb and Zn were lower than in the same species from Karkonosze (SW Poland) reported by Wojtuń et al. (2017) and lower except for Fe than those from unpolluted sites in the Tatra National Park investigated by Šoltés et al. (2014) and Gjengedal et al. (2015). Maximum levels of Fe and Mn were higher and those of Cu and Zn were lower than in this species found 0.5 km from a Cu-Ni smelter (mg kg^-1^): Cu 285, Fe 3896, Zn 205 and Mn 60 (Monni et al. 2002). Remarkable were the concentrations of Mn and Ti in *C. vulgaris* from this investigation as they approached the values typical for toxicity thresholds in plants: 400–1000 and 50–200 mg kg^-1^, respectively (Kabata-Pendias 2001). In spite of the fact that Mn is an essential trace element in plant oxidation and reduction processes, it may be harmful when accumulated in excess (Mousavi et al. 2011; Millaleo et al. 2020). Multiple comparisons of mean ranks showed that *C. vulgaris* contained significantly higher concentrations of Mn than all the other examined species. This is in agreement with Wojtuń et al. (2017) who reported *C. vulgaris* from the Karkonosze with elevated Mn concentrations higher than 400 mg kg^-1^ in shoots with the highest BF for this element compared to the BFs for other elements. Although Mn is most available for plants at low soil pH, in the investigated soil, this factor was close to 7 both in geothermal and control sites. Nonetheless, *C. vulgaris* accumulated very high amounts of Mn. The possible explanation is that *C. vulgaris* possesses long-lived leaves, and therefore, it can accumulate Mn over much longer periods of time compared to other studied species (Losfeld et al. 2015; Kula et al. 2018). Ericaceae typically have ericoid mycorrhizas which can capture Mn (Hashem 1995). This has been reflected in the presented results and applies both to *C. vulgaris* and *E. nigrum* as both belong to the Ericaceae family. Additionally it is known that some species can secrete carboxylates from roots to soil to facilitate P uptake (DeGroote et al. 2018; Lambers et al. 2020). The side effect of this process can be also the increased availability of soil Mn, which enters root cells by nonspecific transporters. It was proved that some Ericaceous species can also release carboxylates (Millaleo et al. 2020). The excessive soil Mn content can alter ability to absorb and accumulate other elements such as Ca, Fe, Mg (Millaleo et al. 2010). The reflection of that statement can be observed in our results as well in the case of Fe accumulation in *C. vulgaris*. Mn is recognised by Millaleo et al. (2010) as an element that concentrates mainly in plant shoots. These findings are in contradiction with the reports that found *C. vulgaris* as a pioneer metallophyte species having an exclusion mechanism (Monni et al. 2000; Salemaa and Uotila 2001; Lottermoser et al. 2011). Because *E. nigrum* also belongs to the Ericaceae family, the reasons mentioned above for *C. vulgaris* also apply to this species, and similar results can be observed. Unlike for *C. vulgaris*, Mn accumulation in *E. nigrum* was clearly observed only in geothermal sites. The maximum concentrations of Cu, Fe and Ni in *E. nigrum* from geothermal sites were higher, and those of Cd, Pb and Zn were lower in comparison with the average levels given by Kabata-Pendias (2001) for terrestrial plants (Table 2). The Ti concentration was within the toxicity thresholds for plants also in this species (Kabata-Pendias 2001). The maximum concentrations of Cd, Cu Fe and Ni in *F. vivipara* from Iceland (Table 3) were higher, and those of Pb and Zn were lower than the average values for terrestrial plants in Table 2 (Kabata-Pendias 2001). Ti concentration in this species was within the toxicity thresholds for plants (Kabata-Pendias 2001). The maximum concentration of Cr was also higher than the harmful levels for plants of 5–30 mg kg^-1^ (Kabata-Pendias 2001). There is no evidence for the indispensability of Cr for plants, but some research proves that small additions of Cr stimulate plant growth and productivity (da Conceição Gomesa et al. 2017). Multiple comparisons of mean ranks showed that *F. vivipara* contained significantly higher concentrations of all the examined elements except for Cu, Mn and Zn than the three other species. The maximum concentration of Cd, Cu, Ni and Pb in *T. praecox* from Iceland was higher for Cu, Fe, Ni and Zn than the average values for terrestrial plants (Tables 2 and 4) reported by Kabata-Pendias (2001). Multiple comparisons of mean ranks showed that *T. praecox* contained significantly higher Zn concentration than *C. vulgaris*, *E. nigrum* and *F. vivipara*. The ability to accumulate very high Zn amounts (up to 50 mg kg^-1^) was found for other species from the Thymus genus, e.g. *T. serpyllum* (Figas et al. 2021). Ti concentration was within the toxicity thresholds for plants also in this species (Kabata-Pendias 2001). Titanium, not considered an essential nutrient in the production of plants and recognised as a potentially harmful metal, may have positive effects (facilitate the uptake of both macro- and micronutrients, increase enzymatic activity and carbohydrate production) when applied at low doses in the so called “hormesis” effect (Markert et al. 2015; Bacilieri et al. 2017). Especially foliar fertilisation can be beneficial because the Ti supply via soil is not effective as the element shows low mobility in soil and limited uptake by roots. The average Ti concentration in terrestrial plants is 0.1–10 mg kg^-1^ (Markert 1992; Tlustoš et al. 2005). The content of this element in plants collected by Ceccantini et al. (1995) from a similar bedrock in Brazil was 1–32 mg kg^-1^. Remarkable was that all the species examined in this investigation contained elevated Ti levels. Tlustoš et al. (2005) believe that Ti accumulates mostly in assimilating tissues, but the usual concentration in plants is rather low. Soils of geothermal fields usually also contain high levels of Ti, among others. This metal is rather mobile in soil solution (Glime 2007; Chiarucci et al. 2008; Wilberscheid 2008; Boothroyda 2009; Lyu et al. 2017) and therefore available for plants by transport through roots (Lyu et al. 2017). Remarkable was also the positive relation between the concentration of Ti available in soil and Ti concentration in *C. vulgaris*, *E. nigrum*, *F. vivipara* and *T. praecox* from geothermal sites*.* ANCOVA showed homogeneity of slopes among these species (F_3,21_ = 0.62, p = 0.610, ESMs 5 and 6). This indicates that the relationships between Ti concentration in soil and that in plants are similar in all examined species. Significant differences in intercepts were found among the species (F_3,24_ =7.64, p < 0.001, ESMs 5 and 6). Multiple comparisons of intercepts among the species showed that the intercept for *F. vivipara* was significantly higher than for *C. vulgaris*, *E. nigrum* and *T. praecox* (ESMs 5 and 6). This means that monocotyledon *F. vivipara* would have the highest concentration of Ti in above-ground parts at any concentration of Ti available in soil in comparison to all the dicotyledonous species examined.

**Table 1:** Minimum, maximum, median and median absolute deviation (MAD) of the concentration (mg·kg^-1^) of metals in *Calluna vulgaris*. P for the U Mann-Whitney test comparing geothermal and control sites. Karkonosze: data from the Karkonosze National Park podzol (Wojtuń et al. 2017). Tatra: data from the Tatra National Park, acid and sandy soils (Šoltés et al. 2014) and organic-rich, podzolic soil (Gjengedal et al. 2015)

	Geothermal				Control						
	Minimum	Maximum	Median	MAD	Minimum	Maximum	Median	MAD	p	Karkonosze	Tatra
As	BDL	3			BDL	BDL					
Cd	0.01	0.06	0.04	0.01	0.001	0.02	0.007	0.01	< 0.01	0.001–0.2	0.1–0.8
Co	0.4	5	1.2	0.8	0.2	0.4	0.3	0.1	< 0.01		
Cr	1.0	12	3.5	2.5	1.9	3	2.3	0.3	NS	0.04–0.3	0.2–6.4
Cu	5.8	17	11	4.1	5.2	7.6	6.6	0.7	< 0.05	5.6–13	4–14
Fe	534	5870	2075	1375	498	930	779	16	< 0.05	49–150	170–710
Hg	0.01	0.03	0.02	0.003	0.005	0.02	0.01	0.009	NS		
Mn	289	659	557	24.7	310	548	520	20.8	NS	68–844	248–880
Ni	3.6	9.5	3.9	0.2	1.4	4.7	3.2	0.7	< 0.05	0.4–5.0	
Pb	0.03	0.3	0.2	0.01	0.1	0.3	0.2	0.01	NS	0.4–3.2	4–9.2
Ti	129	1180	388	234	68	361	233	2.1	NS		
Zn	3.5	9	6.9	1.1	5.7	6.8	6.4	0.3	NS	13–38	28–95

NS = not significant; BDL = detection limit for As < 0.1 mg kg^-1^

**Table 2:** Minimum, maximum, median and median absolute deviation (MAD) of the concentration (mg·kg^-1^) of metals in *Empetrum nigrum*. P for U Mann-Whitney test comparing geothermal and control sites. Plants: average data from Kabata-Pendias (2001) for terrestrial plants

	Geothermal				Control					
	Minimum	Maximum	Median	MAD	Minimum	Maximum	Median	MAD	p	Plants
As	BDL	2.1			BDL	1.2				
Cd	0.01	0.1	0.02	0.01	0.004	0.02	0.006	0.005	< 0.001	< 0.2
Co	0.2	2.7	1	0 .6	0.1	2	0.7	0 .2	NS	
Cr	0.5	13	4.7	2.9	1.1	9.1	2.7	1	NS	
Cu	4	12	6.7	1.8	3.6	8.3	6.6	0.5	NS	< 0.5
Fe	376	3776	1811	940	175	2536	1150	323	NS	< 350
Hg	0.01	1.2	0.03	0.03	0.01	0.02	0.01	0.003	NS	
Mn	169	366	302	50	77	590	136	46	< 0.01	
Ni	2.8	8.7	4 .9	1.1	3.3	8.1	4.9	1.6	NS	< 1
Pb	0.04	0.4	0.1	0 .05	0.1	0.4	0.2	0.08	NS	< 2.5
Ti	106	1482	549	308	35	818	266	155	< 0.05	
Zn	4.4	6.9	5.3	0.7	4.4	19	10	4	< 0.001	< 30

NS = not significant; BDL = detection limit for As < 0.1 mg kg^-1^

**Table 3:** Minimum, maximum, median and median absolute deviation (MAD) of the concentration (mg·kg^-1^) of metals in *Festuca vivipara.* P for U Mann-Whitney test comparing geothermal and control sites

	Geothermal				Control				
	Minimum	Maximum	Median	MAD	Minimum	Maximum	Median	MAD	p
As	BDL	39			BDL	20			NS
Cd	0.03	0.6	0.05	0.01	0.02	0.4	0.06	0.07	NS
Co	0.6	13	4.4	2.7	0.3	16	1.6	1.2	NS
Cr	2.2	53	10.3	7	1.8	60	5.8	3	NS
Cu	3.7	57	10	6.6	4.6	40	6.1	0.6	NS
Fe	1010	15496	5932	3354	456	12817	2163	1436	NS
Hg	0.02	0.06	0.04	0.01	0.01	0.03	0.02	0.005	< 0.05
Mn	107	250	168	38	134	406	217	53	< 0.05
Ni	2.7	23	6.8	3	1.8	45	5.7	3.8	NS
Pb	0.1	1.8	0.3	0.06	0 .04	1.1	0.2	0.1	< 0.05
Ti	236	1868	849	442	105	2307	788	769	NS
Zn	8.1	18	9.4	1.1	7.7	47	15	8.7	< 0.05

NS = not significant

**Table 4:** Minimum, maximum, median and median absolute deviation (MAD) of the concentration (mg·kg^-1^) of metals in *Thymus praecox.* P for U Mann-Whitney test comparing geothermal and control sites. Opole: data for *T. vulgaris* from experimental field in Opole (Rajfur 2015)

	Geothermal				Control					
	Minimum	Maximum	Median	MAD	Minimum	Maximum	Median	MAD	p	Opole
As	BDL	4.7			BDL	1.1				
Cd	0.03	0.1	0.06	0.02	0.01	0.08	0.02	0.003	< 0.001	< 0.7
Co	0.4	6.2	1.4	0.53	0.3	4.2	0.99	0.6	NS	
Cr	0.8	15	2.9	1.7	1.3	6.8	3.3	0.9	NS	
Cu	5.1	16	9.2	3.7	6.3	11	7.7	1.2	NS	< 23
Fe	534	7647	1969	981	405	3576	1358	470	< 0.05	< 821
Hg	0.02	0.1	0.03	0.001	0.01	0.02	0.01	0.002	< 0.01	
Mn	79	236	114	28	44	259	110	30	NS	< 110
Ni	1.5	9.3	2.6	0.9	1	8.5	4.8	1.1	NS	< 34
Pb	0.01	0.6	0.2	0.05	0.001	0.3	0.1	0.1	NS	< 3.5
Ti	181	1496	559	251	64	707	409	144	NS	
Zn	21	42	25	3.1	14	54	28	9	NS	< 25

NS = not significant

The U Mann-Whitney test revealed that *C. vulgaris* from geothermal sites was enriched in Cd, Co, Cu, Fe and Ni; *E. nigrum* was enriched in Cd, Mn and Ti; *F. vivipara* in Hg and Pb; and *T. praecox* in Cd, Fe and Hg in comparison with the same species from the control sites*.* Geothermal fields are usually enriched in various trace elements (Given 1980; Lorenzini 2002; Durowoju et al. 2016). Zn concentration in *E. nigrum* and *F. vivipara* was significantly higher in plants from control sites than from geothermal sites (Tables 2 and 3). This was probably caused by low quantity of organic material in the geothermal soils (Wilberscheid 2008). An increased organic matter level in the soil is related to enhanced Zn availability (Moody et al. (1997).

### Bioaccumulation factor

A bioaccumulation factor (BF) > 1 shows that a species is able to accumulate an element from soil to above-ground tissues (Cluis 2004; Galal and Shehata 2015; Rajfur 2015). All species from both the geothermal and control sites of Iceland had BF < 1 for Cd and Pb (except for *C. vulgaris* from all sites and *F. vivipara* from geothermal sites) (ESMs 7 and 8). This means that the elements were not transferred from soil to above-ground parts of the species (Cluis 2004; Galal and Shehata 2015). Lead is recognised as immobile in alkaline soils thus being not available for plants. However, the acidic to neutral (ESM 3) pH of the examined soils should be favourable for Pb mobility. Both Cd and Pb are toxic for plants (Nagajyoti et al. 2010) and therefore accumulated mainly in roots to protect the photosynthetic apparatus (Kabata-Pendias 2001). Marrs and Bannister (2006) report that the highest concentrations of Pb in *C. vulgaris* were located in the roots and the lowest were in the thin stems and green shoots. BF > 1 for Pb in *C. vulgaris* means that this species is able to accumulate Pb also in shoots (Bartoli et al. 2013). This is in contradiction to Monschein et al. (2010), Lottermoser et al. (2011) and Pippucci et al. (2015) who found *C. vulgaris* as a species with an exclusion mechanism which did not accumulate high levels of metals and thus was able to survive in contaminated mine waste. Various species of Festuca, e.g. *Festuca arundinacea*, *F. vivipara*, *Festuca ovina and Festuca rubra*, have been reported as metal-tolerant with prevailing accumulation in roots (Wong 1982; Brown and Brinkmann 1992; Sigurður and Björn 2005; Szczęśniak 2005; Albornoz et al. 2016; Shabani et al. 2016). Thus, BF > 1 for most of the elements in *F. vivipara* from this investigation shows that the species probably also has accumulation ability in shoots (Fei et al. 2018). *E. nigrum* is recognised as a species with internal metal tolerance (Monni et al. 2000) and accumulates higher metal levels mainly in the old stem tissue when growing in polluted sites (Monni et al. 2002). This investigation also reports *T. vulgaris*, with BF > 1 for most of the elements, as a metal accumulator. The enhanced antioxidant capacity, mainly due to increased accumulation of phenolic compounds, most likely confers protection against the toxic influence of metals to this species (Petrović et al. 2015; Zayova et al. 2018). The highest median BF indicating intensive accumulation for the species growing in geothermal and control areas was found for Ti and Mn for *C. vulgaris*, Ti and Cr for *E. nigrum* and *F. vivipara* and Ti and Zn for *T. praecox.* The highest median BF indicating intensive accumulation for the species growing in geothermal and control areas was found for Ti and Mn for C. vulgaris, Ti and Cr for *E. nigrum* and *F. vivipara* and Ti and Zn for *T. praecox*. Thus, Ti was well accumulated by species belonging to various classes (monocotyledons: *F. vivipara*) and (dicotyledons: *C. vulgaris*, *E. nigrum, T. praecox*) as well as various families: *Ericaceae* (*C. vulgaris*, *E. nigrum*) and *Lamiaceae* (*T. praecox*). The reason why this occurs needs further investigation. According to Kabata-Pendias (2001), Ti is a metal with the lowest bioaccumulation factor in plants contrary to the easily available and mobile Zn. Cook et al. (2009) also report Ti as an element abundant in soil but with low concentrations in plants. Obviously this does not apply to the high Ti concentrations as observed in plants in geothermal areas.

## Conclusion

1. *C. vulgaris* from geothermal sites was enriched in Cd, Co, Cu, Fe and Ni; *E. nigrum* was enriched in Cd, Mn and Ti; *F. vivipara* in Hg and Pb; and *T. praecox* in Cd, Fe and Hg in comparison with the same species from the control sites*.*
2. *C. vulgaris*, *E. nigrum*, *F. vivipara* and *T. praecox* were distinguished by remarkably high concentrations of Ti and *C. vulgaris* also of Mn in amounts typical of toxicity thresholds.
3. Cd and Pb (except for *C. vulgaris* and *F. vivipara*) were not accumulated in the shoots of geothermal plants.
4. *F. vivipara* from geothermal sites had the highest concentration of Ti in above-ground parts at any concentration of plant-available Ti in soil.

## Supplementary Information

ESM 1 (PDF 249 kb)

## Data Availability

The datasets used and/or analysed during the current study are available from the corresponding author on reasonable request.
