# Supplementary material for: Metals in Calluna vulgaris, Empetrum nigrum, Festuca vivipara and Thymus praecox ssp. arcticus in the geothermal areas of Iceland
Source: Environ Sci Pollut Res Int. 2021 Jul 10;28(47):67224–33. doi: 10.1007/s11356-021-15046-3 (PMC8642329; doi:10.1007/s11356-021-15046-3)
Supplement: Supplementary file 1 — (PDF 249 kb) [file 11356_2021_15046_MOESM1_ESM.pdf]

ESM 1.Analysis of Chestnut Soil, Bainaimao and Bayan Obo, Neil Mongol in China GBW07402 (GSS-2) certified reference material

Element	Certified	Found	Recovery	CV	LOD(metod)	LOQ(metod)
	μg·g^{-1}		%		μg·g^{-1}	
As	13.7±1.2	13.5±1.3	98.54	9.6	0.1	0.31
Cd	0.071±0.014	0.069±0.016	104.43	21.9	0.015	0.039
Co	8.7±0.9	8.7±0.7	102.30	7.9	0.025	0.07
Cr	47±4.0	44±4.3	93.62	9.8	0.01	0.033
Cu	16.3±0.9	16.6±1.0	101.84	6.0	0.09	0.32
Fe	40900±700	41600±710	101.71	1.7	40	120
Hg	0.015±0.003	0.0145±0.002	96.67	13.8	0.003	0.01
Mn	510±16.0	508±16.3	99.61	3.2	0.26	1.0
Ni	19.4±1.3	20.1±1.6	103.61	8.0	0,06	0.26
Pb	20±3.0	20.6±2.8	103.00	13.6	0.025	0.085
Ti	2710±80	2705±85	99.82	3.1	0.3	1.0
Zn	42±3.0	42.6±3.6	101.43	8.5	0.16	0.5

ESM 2.Analysis of *Poaceae (mixture)* IPE 952WEPALcertified reference material

Element	Certified	Found/Recovered	Recovery	CV	LOD(metod)	LOQ(metod)
	μg·g^{-1}		%		μg·g^{-1}	
As	0.298±0.007	0.290±0.009	97.32	3.1	0.1	0.31
Cd	2.91±0.08	2.85±0.07	97.94	2.5	0.015	0.039
Co	0.273±0.01	0.280±0.02	102.56	7.1	0.01	0.041
Cr	3.69±0.07	3.65±0.08	98.92	2.2	0.01	0.033
Cu	14.5±0.13	14.9±0.11	102.76	0.7	0.02	0.058
Fe	493±19	499±18	101.22	3.6	0.7	2.4
Hg	0.0339±0.02	0.0441±0.01	98.82	29.6	0.003	0.01
Mn	81.5±1.4	83.1±1.2	101.99	1.4	0.18	0.55
Ni	6.74±0.07	6.77±0.05	100.45	0.7	0,06	0.26
Pb	6.55±0.22	6.59±0.24	100.61	3.6	0.025	0.085
Zn	82±0.33	84±0.39	102.44	0.5	0.12	0.4

ESM 3. Minimum, maximum, median and median ± absolute deviation (MAD) of soil temperature (T), altitude (A), soil pH as well as the total concentration (Fe g/kg^{-1}, all others mg·kg^{-1}) of metals in soil. P for the U Mann-Whitney test for the comparison of geothermal and control sites. The column Iceland shows total concentration (mg·kg^{-1}) of metals in soils of this island as reported by Kolon et al. (2020)

	Geothermal			Control				
	Minimum	Maximum	Median ± MAD	Minimum	Maximum	Median ± MAD	p	Iceland
T	32	59	40 ± 4	11	25	20 ± 4.0	<0.001	
A	69	635	344 ± 137	61	646	304 ± 116	NS	
pH	5.6	7.1	6.9 ± 0.3	6.1	7.1	6.5 ± 0.2	<0.01	
As	4.7	37	18 ± 5.6	4.5	24	10 ± 8.1	<0.05	
Cd	0.05	0.2	0.07 ± 0.03	0.06	0.6	0.12 ± 0.04	<0.05	<0.5
Co	14	52	34 ± 12	13	69	32 ± 16	NS	<49
Cr	18	188	51 ± 26	16	105	33 ± 16	<0.05	<96
Cu	42	167	88 ± 41	39	169	76 ± 37	NS	<113
Fe	30	121	99 ± 14	27	118	59 ± 29	<0.01	<86
Hg	0.01	1.2	0.875 ± 0.24	0.01	0.2	0.011 ± 0.001	<0.001	<0.3
Mn	482	1659	1187 ± 479	410	1789	845 ± 385	NS	<1321
Ni	26	95	51 ± 16	21	97	42 ± 20	NS	<119
Pb	1.4	18	5.9 ± 3.1	0.5	16	2.9 ± 2.2	NS	<48
Ti	2040	9785	4410 ± 548	857	4610	3040 ± 1390	<0.01	
Zn	40	195	138 ± 37	32	150	114 ± 22	<0.01	<179

ESM 4. Minimum, maximum, median and median absolute deviation (MAD) of the plant-available concentration ($mg \cdot kg^{-1}$) of metals in soil. P for the U Mann-Whitney test for the comparison of geothermal and control sites. The column Iceland shows the plant-available elements ($mg \cdot kg^{-1}$) in non-geothermal soils of this island as reported by Kolon et al. (2020)

	Geothermal					Control					
	Minimum	Maximum	Median	MAD		Minimum	Maximum	Median	MAD	p	Iceland
As	0.1	1.9	0.4	0.2	<	0.4	1.9	0.7	0.2	<0.001	
Cd	0.001	0.06	0.02	0.01		0.001	0.1	0.02	0.02	NS	<0.2
Co	0.6	2.9	0.9	0.3		0.4	1.3	0.9	0.2	NS	<4.0
Cr	0.1	0.6	0.4	0.1		0.2	0.8	0.3	0.1	NS	<1.0
Cu	2.8	14	4.7	0.9	<	3.1	24	6.0	3.0	<0.05	<20
Fe	261	580	423	76	<	305	642	475	64	NS	<1542
Mn	29	509	44	32	>	16	209	33	52	<0.05	<7.5
Ni	0.2	1.3	0.4	0.1	<	0.3	5.5	0.8	0.7	<0.05	<12
Pb	0.03	0.8	0.2	0.2		0.03	1.5	0.3	0.4	NS	<40
Ti	6.9	65	29	7.6	<	26	66	39	5.5	<0.01	
Zn	1.3	4.5	1.7	0.3		0.9	11	1.9	0.5	NS	<40

ESM 5. Slope b± SE (Standard Error) and intercept a± SE (Standard Error) values, percentage of variance explained (R2) and significance (p) for regression analyses relating Ti concentration in a species to Ti concentration available in soil. Intercepts significantly different from zero are indicated in boldface. Sample size (n) in each species is indicated in the last column

Species	b ± SE	a ± SE	R^2	p	n
Calluna vulgaris	24.7 ± 5.5	-118.2 ± 159.3 β	0.83	<0.01	6
Empetrum nigrum	17.0 ± 4.0	22.3 ± 123.7 b	0.78	<0.01	7
Festuca vivipara	17.2 ± 4.5	**411.9**± 145.1 a	0.83	<0.01	8
Thymus praecox	16.4 ±2.9	175.9 ± 91.6 b	0.84	<0.01	8

[1] The same letter indicates that values were not significantly different (Tukey test)

ESM. 6. Relationship between Ti concentration (mg kg^{-1}) in *Calluna vulgaris* (blue, circle), *Empetrum nigrum* (red, square), *Festuca vivipara* (violet, triangle) and *Thymus praecox* (green, diamond) and the concentration of plant-available Ti (mg kg^{-1}) in soil

Ti *C.vulgaris* = -118.209+24.7006*x

Ti *E. nigrum* = 22.3489+16.9848*x

Ti *F. vivipara* = 411.9382+17.2656*x

Ti *T. praecox* = 175.968+16.4052*x

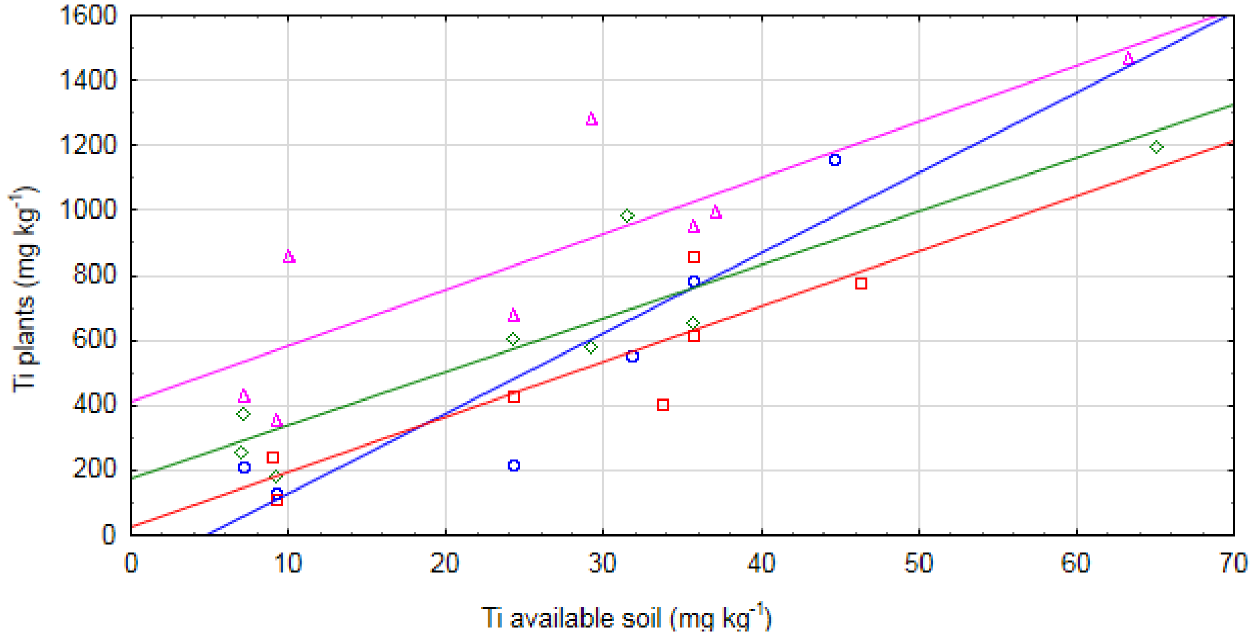

ESM 7. Minimum (Min), maximum (Max), and median (Me) of the Bioaccumulation Factor BF (plant-to-soil plant-available metal concentration ratio) in *Calluna vulgaris* (BF*Cv*), *Empetrum nigrum* (BF*En*), *Festuca vivipara* BF*Fv*), *Thymus praecox* (BF*Tp*) from geothermal sites

	BF*Cv*			BF*En*			BF*Fv*			BF*Tp*		
	Min	Max	Me	Min	Max	Me	Min	Max	Me	Min	Max	Me
Cd	0.002	0.4	0.03	0.002	0.08	0.02	0.006	0.6	0.06	0.004	0.6	0.04
Co	0.2	6.7	1.9	0.1	3.7	1.5	0.4	9.7	2.8	0.4	8.4	1.1
Cr	1.7	37	12	1.2	40	17	3.6	175	34	1.8	45	12
Cu	0.5	3.4	2.6	0.4	2.4	1.6	0.3	5.9	1.9	0.5	3.9	1.6
Fe	1.4	12	5.3	1.4	7.7	4.2	1.7	38	12	2.2	16	5.4
Mn	0.8	37	14	2.4	22	7.9	0.4	15	2.8	0.3	8.1	2.5
Ni	3.0	25	7.0	3.3	23	11	3.4	47	12	1.2	27	5.4
Pb	0.08	9.4	1.7	0.3	2.9	0.8	0.3	7.0	2.3	0.05	5.9	0.8
Ti	14	26	20	8.8	33	15	19	77	36	18	52	22
Zn	1.4	6.6	3.8	1.5	4.2	2.9	2..7	11	5.6	7.5	25	14

ESM 8. Minimum (Min), maximum (Max), and median (Me) of the Bioaccumulation Factor BF (plant-to-soil plant-available metal concentration ratio) in *Calluna vulgaris* (BF*Cv*), *Empetrum nigrum* (BF*En*), *Festuca vivipara* (BF*Fv*), *Thymus praecox* (BF*Tp*) from control sites.

	BF*Cv*			BF*En*			BF*Fv*			BF*Tp*		
	Min	Max	Me	Min	Max	Me	Min	Max	Me	Min	Max	Me
Cd	0.0004	0.01	0.002	0.001	0.03	0.003	0.001	0.2	0.03	0.002	0.5	0.008
Co	0.1	0.4	0.3	0.07	1.8	1.0	0.4	27	1.4	0.3	4.9	1.1
Cr	2.3	10	5.0	1.3	16	10	2.1	216	16	2.8	26	7.2
Cu	1.1	1.4	1.3	0.3	2.3	0.8	0.2	5.4	0.9	0.3	1.9	1.1
Fe	1.2	2.1	1.8	0.4	5.4	1.8	1.1	25	3.7	0.9	5.9	2.5
Mn	9.6	17	16	1.0	16	3.6	0.7	17	4.8	0.6	5.8	1.5
Ni	2.0	5.6	3.9	1.3	18	3.2	1.1	33	3.4	0.6	9.2	1.5
Pb	0.5	3.5	1.6	0.05	4.9	0.5	0.03	9.3	0.3	0.002	2.9	0.4
Ti	1.7	10	6.3	3.1	28	7.7	2.6	36	20	1.6	18	9.7
Zn	2.8	4.0	3.3	0.9	14	6.1	1.1	14	5.8	4.2	21	11
